# Osteoporosis in patients with rheumatoid arthritis: trends in the German National Database 2007–2017

**DOI:** 10.1007/s00296-020-04593-6

**Published:** 2020-05-06

**Authors:** Lisa Lindner, Johanna Callhoff, Rieke Alten, Andreas Krause, Wolfgang Ochs, Angela Zink, Katinka Albrecht

**Affiliations:** 1 Epidemiology Division, German Rheumatism Research Center (DRFZ), Charitéplatz 1, 10117 Berlin, Germany; 2 Schlosspark Klinik, Internal Medicine 2, Rheumatology, Clinical Immunology and Osteology, Berlin, Germany; 3 Immanuel Hospital, Rheumatology and Clinical Immunology, Berlin, Germany; 4 Rheumatism Practice Bayreuth, Bayreuth, Germany; 5 Medical Clinic with Focus on Rheumatology and Clinical Immunology, Charité – Universitätsmedizin, Berlin, Germany

**Keywords:** Health care provision, Comorbidity, Glucocorticoids, Disease activity, Remission

## Abstract

Osteoporosis is a frequent comorbidity in rheumatoid arthritis (RA). Due to the improved treatment options for RA, we expect a long-term decrease in osteoporosis as an accompanying disease. Data from the German National Database (NDB) were used to investigate whether the frequency of osteoporosis has changed in the last 10 years. From 2007 to 2017, approximately 4000 patients were documented annually with data on therapy and comorbidity. The cross-sectional data were summarised descriptively. Age, sex, disease duration, disease activity and glucocorticoids were considered as influencing factors. The Cochrane-Armitage test for trend was used to test whether the frequency of osteoporosis at the first visit changed from 2007 to 2017. Osteoporosis frequency in RA patients (mean age 63 years, 75% female) decreased from 20% in 2007 to 6% in 2017 (*p* < 0.001). The decrease affected women (22% to 17%) and men (14% to 8%) in all age groups and both short-term (≤ 2-year disease duration: 9% to 3%) and long-term RA patients (> 10-year disease duration: 28% to 20%). Patients with high disease activity and patients who took glucocorticoids (GC) were more often affected by osteoporosis than patients in remission or without GC. Drug prophylaxis in patients without osteoporosis increased (20% to 41% without GC, 48% to 55% with GC). Men with GC received less prophylactic treatment than women (48% vs. 57% in 2017). In this cohort, osteoporosis in patients with RA is less frequently observed compared to former years. RA-specific risk factors for osteoporosis such as disease activity and GC therapy have declined but long-term GC use is still present. Assessment of osteoporosis in RA patients should be investigated more consistently by bone density measurement. Male RA patients still need to be given greater consideration regarding osteoporosis drug prophylaxis, especially when GC therapy is needed.

## Introduction

Rheumatoid arthritis (RA) is often accompanied by other diseases. The prognosis and course of RA can be significantly influenced by comorbidities and therapeutic options are often limited by comorbidities. Comorbidities in RA are associated with inflammatory activity, disease-related organ damage or medication [1]. They may be present before the onset of RA, develop during the course of the disease, associated with RA or iatrogenically induced [2]. Osteoporosis is a very common comorbidity in RA [3]. It is a strong risk factor for fractures due to the loss of bone density. In a population cohort of 47,000 RA patients, the risk of osteoporotic fractures was about 1.5 times as high as in people without RA [4]. Osteoporosis-related fractures can contribute to loss of function, increased morbidity and mortality and rising health care costs [5–7]. In addition to the general risk factors of osteoporosis, RA patients face additional risks if inflammatory activity is not sufficiently controlled and glucocorticoid therapy is needed.

The treatment of RA and the therapeutic options have improved significantly in recent years. This is mainly due to a better understanding of pathogenic mechanisms and the development of new drugs [8, 9]. Therefore, we can expect RA-associated osteoporosis to decrease with good disease control of RA.

In this context, possible changes in the frequency of osteoporosis in RA patients over the last 10 years were investigated. The data from the National Database of the German Collaborative Arthritis centers (NDB) allow investigating trends over a long period of time with a comparable patient collective. The research question of the study was whether RA-specific risk factors and the frequency of osteoporosis have changed in the last decade.

## Patients and methods

In the NDB, data are continuously collected from rheumatological patient care. Every year, rheumatologist- and patient-reported data are collected [10]. The data on which this evaluation is based derive from 11 rheumatologist practices and 8 rheumatology centres.

For the present analysis, all patients with RA and available data on treatments and comorbidities were included. Cross-sectional data are presented from 2007 to 2017. The following patient parameters were considered for each year: age, sex, body mass index (BMI in kg/m^2^). Functional limitation was assessed by the Hannover Functional Ability Questionnaire (FFbH), being transformed into health assessment questionnaire values (HAQ, 0 to 3 with 0 representing full capacity) [11]. The disease-specific parameters and details of the therapy were documented by the rheumatologist. These are onset of symptoms, disease activity, measured by the Disease Activity Score (DAS28), a set of 20 comorbidities (yes/no) and therapy information on glucocorticoids (GC), GC dose in mg prednisone equivalent per day, non-steroidal anti-inflammatory drugs (NSAIDs), conventional synthetic disease-modifying antirheumatic drugs (csDMARDs), biologic (b)DMARDs, medical osteoporosis prophylaxis (e.g. calcium and vitamin D) and osteoporosis therapy (e.g. bisphosphonates but this is not further specified in the questionnaire). With regard to osteoporosis, rheumatologists indicate whether osteopenia, osteoporosis or osteoporosis with pathological fracture is present (the appropriate one is ticked). No definition according to dual-energy X-ray absorptiometry (DXA) criteria is required, but the definition of osteoporosis is within the responsibility of the rheumatologist. When marking osteopenia, it can be assumed that the DXA findings (*T* < − 1 and > − 2.5) are used as a criterion. Further comorbidities of RA are also reported as present or not: degenerative joint disease, heart–lung disease, diabetes mellitus, thyroid disease, lipid metabolism disorder and kidney disease. Furthermore, it is queried whether a bone density measurement was carried out in the last 12 months, respectively, ever.

### Statistical analysis

Descriptive statistics (mean, standard deviation (SD) and percentages) were used to summarise patients’ characteristics, disease activity, treatments and osteoporosis comorbidity for each calendar year. To test whether the frequency of osteoporosis at the first visit changed over time, osteoporosis frequencies from 2007 to 2017 were compared with the Cochrane–Armitage test for trend.

### Ethics approval

The database received study approval from the ethics committee of the Charité—Universitätsmedizin Berlin (EA1/196/06) in February 2007.

## Results

### Patient characteristics

From 2007 to 2017, between 3500 and 5500 RA patients per year were included. Over the years, the proportion of female patients (75%), smoking (20%), average BMI (26 kg/m^2^) and the proportion of underweight patients (BMI < 18.5: 2%) remained constant. The mean age (62 to 63 years) and disease duration (12 to 14 years) increased by 1.5–2 years (Table 1).

**Table 1:** Patient characteristics

	2007	2008	2009	2010	2011	2012	2013	2014	2015	2016	2017
*N*	3576	4243	4566	5338	5504	5019	5069	4902	5197	5473	5423
Female, %	76	75	75	75	75	76	76	75	76	75	75
Age in years, mean (SD)	62 (13)	62 (12)	62 (13)	62 (13)	62 (13)	63 (13)	63 (14)	63 (14)	63 (14)	63 (14)	63 (14)
Disease duration in years, mean (SD)	12 (11)	12 (10)	12 (10)	13 (10)	13 (10)	13 (10)	13 (10)	14 (10)	14 (11)	14 (11)	14 (11)
Current smoking, %	19	19	18	20	20	20	20	20	21	20	21
Body mass index in kg/m^2^, mean (SD)	26,4 (4,9)	26,6 (4,9)	26,6 (4,9)	26,6 (5,0)	26,5 (5,0)	26,6 (5,0)	26,6 (4,9)	26,3 (4,9)	26,3 (5,0)	26,4 (5,0)	26,3 (5,0)

*SD* standard deviation

### RA disease activity and functional status

The mean disease activity of RA, measured by DAS28, decreased from 3.1 to 2.8 (Table 2). The proportion of patients with moderate to high disease activity (DAS28 > 3.2) decreased from 41 to 29% while the proportion of patients in remission increased from 38 to 53%. The proportion of patients with clinically relevant functional impairment, measured by the HAQ > 1.5 decreased from 31 to 25% (Table 2).

**Table 2:** Trends in RA-specific characteristics and osteoporosis

	2007	2008	2009	2010	2011	2012	2013	2014	2015	2016	2017
DAS-28, mean	3,1	3,1	2,9	2,9	2,9	2,9	2,9	2,8	2,7	2,7	2,8
≤ 2.6, %	38	40	46	45	45	47	47	50	51	53	53
2.6–3.2, %	21	21	20	20	19	20	20	20	21	19	17
3.2–5.1, %	22	20	18	18	19	19	19	18	16	16	26
> 5.1 %	19	20	16	17	17	14	15	13	12	11	3
HAQ > 1.5, %	31	30	30	30	28	27	28	27	26	26	25
Treatment											
csDMARDs, %	71	69	66	66	64	63	62	61	61	59	58
bDMARDs, %	18	20	23	22	24	26	27	27	28	28	27
NSAIDS, %	41	37	42	43	39	38	39	41	42	38	35
GCs, %	60	58	57	55	52	52	50	48	47	45	43
Thereof > 7.5 mg/d, %	16	14	14	14	13	13	12	14	13	13	14
Duration of intake of GCs in months, mean (SD)	61 (79)	59 (76)	61 (78)	59 (78)	59 (77)	61 (78)	62 (80)	59 (80)	58 (79)	58 (78)	58 (79)

csDMARDs conventional synthetic disease-modifying anti-rheumatic drug; DAS28 Disease activity Score; HAQ Health Assessment Questionnaire, 0 representing full functional capacity; GC glucocorticoids, NSAIDS non-steroidal anti-inflammatory drugs; SD standard deviation

### Treatments

Treatment with bDMARDs has increased from 18 to 27% while csDMARDs were used less often (71% to 58%). There was a significant decrease in the use of GCs (60% to 43%) but the proportion of patients with GC doses above 7.5 mg/d only decreased slightly (16% to 14%) and the mean duration of GC use remained 5 years. The proportion of patients who received NSAIDs remained stable (~ 40%).

### Osteoporosis

From 2007 to 2017, the frequency of osteoporosis including patients at first visit decreased from 20% to 6% (*p* < 0.001). The proportion of patients with osteopenia remained at 5%. Pathological fractures were documented in about 4% of patients who had osteoporosis. Other comorbidities (e.g. degenerative joint diseases, heart/lung diseases) did not decrease (Table 3). Overall, the number of patients with ≥ 3 comorbidities increased (36% to 44%).

**Table 3:** Osteoporosis and other comorbidities

	2007	2008	2009	2010	2011	2012	2013	2014	2015	2016	2017
Osteopenia	–	–	4	3	4	5	5	5	5	4	4
Osteoporosis	20	18	18	17	17	18	17	16	16	15	15
Thereof with fracture	–	–	3	3	4	4	4	4	4	4	3
Degenerative joint disease	16	18	18	20	19	21	23	22	21	20	20
Heart diseases	13	11	12	12	12	12	11	12	11	11	11
Chronic lung disease	9	9	8	9	10	10	10	11	11	10	11
Diabetes mellitus	11	10	11	12	11	11	11	12	11	11	10
Kidney disease	6	6	7	7	7	7	7	7	7	6	6
Fat metabolism disorder	9	9	11	11	11	11	11	11	10	10	10
≥ 3 comorbidities	36	35	41	44	44	46	47	48	46	45	44

All numbers are percentages

**Fig. 1:**
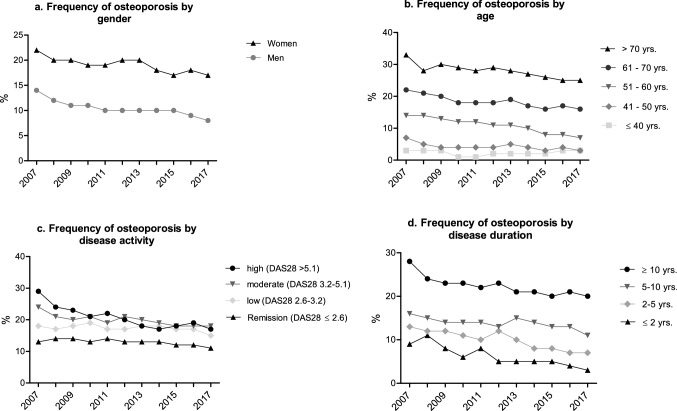
Frequency of osteoporosis by **a** sex, **b** age, **c** disease activity and **d** disease duration

Osteoporosis was documented more frequently in women than in men and more often in older age groups (Fig. 1a, b). A decrease was observed irrespective of age and sex. Osteoporosis was frequent in patients with high disease activity (according to DAS28) and rare in patients in remission (Fig. 1c). Patients in remission had a consistently low osteoporosis rate of 12.8% over the years. In the group with medium and high disease activity, a declining trend was observed.

Osteoporosis was most frequent in patients with RA disease duration ≥ 10 years and lowest in those with a disease duration of 2 years or less. For all groups stratified by disease duration, the frequency of osteoporosis decreased, most markedly in the group with disease duration of more than 10 years (Fig. 1d).

One in five patients under GC therapy was reported to have osteoporosis, and the proportion decreased only slightly (22% in 2007 and 19% in 2017). In patients without GC therapy, osteoporosis was present in 12%. Osteoporosis was more common in patients with bDMARDs (18.5%) than in patients who did not receive bDMARDs (16.3%).

### Osteoporosis prophylaxis and therapy

**Fig. 2:**
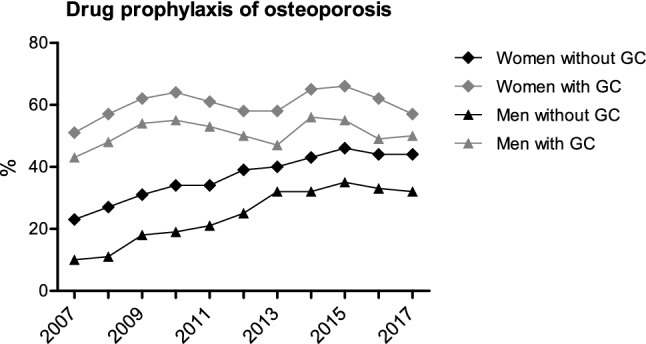
Drug prophylaxis of osteoporosis (%)

Drug prophylaxis in patients without osteoporosis increased (20% to 41% without GC, 48% to 55% with GC). Under GC therapy, women and men received osteoporosis prophylaxis more frequently (Fig. 2). Males with GC received prophylactic treatment less frequently than women (48% vs. 57% in 2017). However, the increase in prophylaxis was more pronounced for patients without GC (females 24% to 42% and males 10% to 31%).

### Bone density measurement

The frequency of bone density measurements in the last 12 months before recruitment decreased and in 2017, a measurement was reported in only 5% of patients. The frequency of DXA ever performed increased, but remained below 50% until 2017. Bone density measurements were less frequently performed in younger age groups than in patients > 60 years (Table 4).

**Table 4:** Osteoporosis treatments and bone density measurement

	2007	2008	2009	2010	2011	2012	2013	2014	2015	2016	2017
Drug prophylaxis (e.g. calcium + Vitamin D)	37	42	46	47	45	46	47	51	53	49	47
Treatment (e.g. bisphosphonates)	18	16	19	20	18	18	18	18	17	14	13
Bone densitometry in the last 12 months	17	13	15	14	14	11	11	11	10	9	5
≤ 40 years	6	3	7	6	7	5	2	4	3	2	2
41–50 years	13	9	9	11	9	8	9	5	4	4	2
51–60 years	15	12	13	13	11	10	9	8	8	7	4
61–70 years	18	16	18	15	15	13	13	15	10	11	7
> 70 years	22	16	16	17	18	13	13	12	14	13	6
Bone densitometry ever	26	19	32	34	36	40	42	43	43	40	41

All numbers are percentages

## Discussion

In the 10-year trends from the NDB, we observe a decline of osteoporosis as a concomitant disease of RA compared to other comorbidities among patients treated in the participating institutions. The distribution of general risk factors for the development of osteoporosis recorded in the NDB, such as age, sex, smoking and underweight, did not change during this period. The more frequent occurrence of osteoporosis in women and at an older age is well known, not RA specific and not modifiable. On the other hand, the connection between osteoporosis and disease activity in RA is of great importance. Due to the improved treatment options over the last 10 years, we now see more RA patients in remission or low disease activity. Compared to patients with high disease activity, they are less often reported to have osteoporosis. Lower frequency of osteoporosis and lower disease activity in later years could be attributed to treatment improvements and as a consequence of lower disability and higher physical activity. More frequent osteoporosis with increasing disease duration of RA can also be observed. However, statements about a causal relationship cannot be made due to the cross-sectional design. Innala et al. [12] showed a clear correlation between disease duration and osteoporosis incidence in their study population. They determined the prevalence at inclusion and after 5 years in the same patients. After 5 years of illness, the incidence of osteoporosis had more than doubled (1.4% vs. 3.7%). It could be assumed that due to the increasing disease duration of RA in the NDB, osteoporosis is also more frequent over the years. In fact, we observe a decrease over time suggesting that there are other important causal factors for this decrease.

In addition to disease activity, long-term or higher dose GC therapy plays an important role. In our data, patients with GC have osteoporosis 1.5 times as often as patients without GC. If patients can be brought into GC-free remission, osteoporosis may be reduced to a level comparable to that in the general population as it has been shown that patients in remission have a similar risk profile for the development of osteoporosis as the general population [13]. The perception of the high risk of osteoporosis in RA resulting from both inflammatory activity and GC therapy has increased in recent years. In addition to the decline in GC therapies, we also see an increase in osteoporosis drug prophylaxis in RA patients without osteoporosis. Various studies have shown inadequate osteoporosis medication in RA [14]. In a retrospective cohort study [15], 38% of GC users were treated with osteoporosis drugs, 57% of women and only 9% of men. Especially men and premenopausal women remained undersupplied. The situation is similar for prophylactic treatment of GC patients. In the NDB, about 60% of patients with GCs receive osteoporosis prophylaxis. For women, the proportion has doubled, for men even tripled. This could be an indication that sensitivity to the risk of osteoporosis in men has increased in recent years. Another reason could be that the patients included in the NDB are getting older, thus have a higher risk profile and receive appropriate prophylactic treatment in accordance with German and international guidelines [16, 17].

However, the long-term GC use and the remaining high GC doses in more than 10% of RA patients are risk profiles that need to be modified with today’s treatment options. It is known that the duration of GC intake can cause increased bone density loss. Van Staa et al. [18] reported that the risk of fracture decreases immediately after discontinuation of GC. In addition to the duration, the dose also affects bone density loss. A meta-analysis [19] demonstrated the positive correlation between cumulative GC dose and low bone mass. Besides GC reduction, bone density measurement should be given a higher priority in the routine diagnosis of RA. The documented examinations in the NDB are insufficient and confirm that osteoporosis is not examined consistently enough by means of bone density measurement even in rheumatism centres [3]. However, we assume that some of the bone density measurements performed were not documented. Studies that measure the prevalence of osteoporosis using DXA measurements show significantly higher frequencies [20] than our analysis. Nationwide claims data from the PROCLAIR study also show a significantly higher frequency of osteoporosis diagnosis compared to the NDB, which is 2.8 times as high as in persons with RA compared to the general population [21]. In the early arthritis cohort CAPEA, the frequency of osteoporosis after a 2-year observation period was 25% in women and 15% in men (mean age women 55 years, men 58 years) [22]. In CAPEA, after 2 years of observation, the values of bone density measurements were explicitly asked for, so that these patients may have been examined more frequently.

### Limitations

The presence of osteoporosis is based on information from rheumatologists, not on systematic diagnostic examinations. However, our study lacks data on many traditional risk factors for osteoporosis, especially information on the contribution of menopause to bone loss. On the basis of the NDB, it is not possible to evaluate how many patients have an indication for bone density measurement. Also, low frequencies of BMD measurement especially in patients aged > 50 years in 2016 and 2017 may have led to an underestimation of osteoporosis in RA patients in later groups. The cross-sectional analyses cannot make any statements about temporal relationships between exposure and outcome. Furthermore, under-recording and undiagnosed comorbidities can represent a limitation since no further diagnostic criteria were queried. The presence of degenerative disease in > 20% of patients in later groups vs. less than 18% in 2007–2010 may also be a cause for underestimating osteoporosis, particularly lumbar spine osteoporosis. Analyses involving the GC dose could not be performed due to the cross-sectional design. The reason for this is that the available cross-sectional data cannot provide any information about the history of therapy, i.e. previous or cumulative doses.

## Conclusion

In this cohort, osteoporosis in patients with RA is less frequently observed compared to former years. RA-specific risk factors for osteoporosis such as disease activity and GC therapy have declined but long-term GC use is still present.

Assessment of osteoporosis in RA patients should be investigated more consistently by bone density measurement. Male RA patients still need to be given greater consideration regarding osteoporosis drug prophylaxis, especially when GC therapy is needed.

## References

[1] Sokka T, Puolakka K, Turesson C (2013) Comorbidities of rheumatic disease. Oxford Textbook of Rheumatology 243

[2] Albrecht K (2014). Comorbidity in rheumatoid arthritis. Dtsch Med Wochenschr.

[3] Gaubitz M (2019). Osteoporosis-frequent comorbidity in patients with rheumatism. Z Rheumatol.

[4] Kim SY, Schneeweiss S, Liu J, Daniel GW, Chang CL, Garneau K, Solomon DH (2010). Risk of osteoporotic fracture in a large population-based cohort of patients with rheumatoid arthritis. Arthritis Res Ther.

[5] Bleibler F, Rapp K, Jaensch A, Becker C, Konig HH (2014). Expected lifetime numbers and costs of fractures in postmenopausal women with and without osteoporosis in Germany: a discrete event simulation model. BMC Health Serv Res.

[6] Mau W, Thiele K, Lamprecht J (2014). Trends of work force participation of patients with rheumatic diseases: results from German social insurance data and the national database of the German collaborative arthritis centers. Z Rheumatol.

[7] Widdifield J, Paterson JM, Huang A, Bernatsky S (2018). Causes of death in rheumatoid arthritis: how do they compare to the general population?. Arthritis care & research.

[8] Kalden JR (2016). Emerging therapies for rheumatoid arthritis. Rheumatol Ther.

[9] Smolen JS, Breedveld FC, Burmester GR, Bykerk V, Dougados M, Emery P, Kvien TK, Navarro-Compan MV, Oliver S, Schoels M, Scholte-Voshaar M, Stamm T, Stoffer M, Takeuchi T, Aletaha D, Andreu JL, Aringer M, Bergman M, Betteridge N, Bijlsma H, Burkhardt H, Cardiel M, Combe B, Durez P, Fonseca JE, Gibofsky A, Gomez-Reino JJ, Graninger W, Hannonen P, Haraoui B, Kouloumas M, Landewe R, Martin-Mola E, Nash P, Ostergaard M, Ostor A, Richards P, Sokka-Isler T, Thorne C, Tzioufas AG, van Vollenhoven R, de Wit M, van der Heijde D (2016). Treating rheumatoid arthritis to target: 2014 update of the recommendations of an international task force. Ann Rheum Dis.

[10] Albrecht K, Callhoff J, Zink A (2019). Long-term trends in rheumatology care: achievements and deficits in 25 years of the German national rheumatology database. Z Rheumatol.

[11] Lautenschlager J, Mau W, Kohlmann T, Raspe HH, Struve F, Bruckle W, Zeidler H (1997). Comparative evaluation of a German version of the Health Assessment Questionnaire and the Hannover Functional Capacity Questionnaire. Z Rheumatol.

[12] Innala L, Sjoberg C, Moller B, Ljung L, Smedby T, Sodergren A, Magnusson S, Rantapaa-Dahlqvist S, Wallberg-Jonsson S (2016). Co-morbidity in patients with early rheumatoid arthritis—inflammation matters. Arthritis Res Ther.

[13] Ajeganova S, Huizinga T (2017). Sustained remission in rheumatoid arthritis: latest evidence and clinical considerations. Ther Adv Musculoskelet Dis.

[14] Wen L, Kang JH, Yim YR, Lee JW, Lee KE, Park DJ, Kim TJ, Park YW, Lee SS (2016). Risk factors for treatment failure in osteoporotic patients with rheumatoid arthritis. Mod Rheumatol.

[15] Feldstein AC, Elmer PJ, Nichols GA, Herson M (2005). Practice patterns in patients at risk for glucocorticoid-induced osteoporosis. Osteoporosis Int.

[16] Pfeil A, Lehmann G, Lange U (2018). Update DVO guidelines 2017 on “Prophylaxis, diagnostics and treatment of osteoporosis in postmenopausal women and men”: what is new, what remains for rheumatologists?. Z Rheumatol.

[17] Buckley L, Guyatt G, Fink HA, Cannon M, Grossman J, Hansen KE, Humphrey MB, Lane NE, Magrey M, Miller M, Morrison L, Rao M, Byun Robinson A, Saha S, Wolver S, Bannuru RR, Vaysbrot E, Osani M, Turgunbaev M, Miller AS, McAlindon T (2017). 2017 American College of Rheumatology Guideline for the Prevention and Treatment of Glucocorticoid-Induced Osteoporosis. Arthritis Care Res (Hoboken).

[18] van Staa TP, Leufkens HG, Abenhaim L, Zhang B, Cooper C (2000). Oral corticosteroids and fracture risk: relationship to daily and cumulative doses. Rheumatology (Oxford, England).

[19] van Staa TP, Leufkens HG, Cooper C (2002). The epidemiology of corticosteroid-induced osteoporosis: a meta-analysis. Osteoporosis Int.

[20] Hauser B, Riches PL, Wilson JF, Horne AE, Ralston SH (2014). Prevalence and clinical prediction of osteoporosis in a contemporary cohort of patients with rheumatoid arthritis. Rheumatology (Oxford, England).

[21] Luque Ramos A, Redeker I, Hoffmann F, Callhoff J, Zink A, Albrecht K (2019). Comorbidities in patients with rheumatoid arthritis and their association with patient-reported outcomes: results of claims data linked to questionnaire survey. J Rheumatol.

[22] Albrecht K (2014). Gender-specific differences in comorbidities of rheumatoid arthritis. Z Rheumatol.

